# Hidden Metabolic Disturbances in Women with Normal Glucose Tolerance Five Years after Gestational Diabetes

**DOI:** 10.1155/2015/342938

**Published:** 2015-03-22

**Authors:** Yvonne Winhofer, Andrea Tura, Anita Thomas, Thomas Prikoszovich, Christine Winzer, Giovanni Pacini, Anton Luger, Alexandra Kautzky-Willer

**Affiliations:** ^1^Division of Endocrinology and Metabolism, Department of Internal Medicine III, Medical University of Vienna, Waehringer Guertel 18-20, 1090 Vienna, Austria; ^2^Metabolic Unit, Institute of Biomedical Engineering, National Research Council, 35127 Padova, Italy; ^3^Division of Nephrology, Department of Internal Medicine III, Medical University of Vienna, 1090 Vienna, Austria

## Abstract

*Background*. The study aimed to assess whether women with prior gestational diabetes (pGDM), despite maintenance of normal glucose tolerance (NGT) five years after delivery, display metabolic disturbances compared to healthy controls. *Methods*. 45 pGDM with NGT were compared to 18 women without a history of GDM (CON), matched for age (37.0 ± 4.1 versus 35.2 ± 5.3, *P* = ns) and BMI (24.3 ± 3.1 versus 23.3 ± 3.3, *P* = ns). Metabolic parameters were derived from oral and intravenous glucose tolerance tests; furthermore lipid profile, C-reactive protein (CRP), adiponectin, leptin, and glucagon were assessed. *Results*. Five years postpartum, pGDM had increased glucose concentrations during the OGTT (AUC: 1.12 ± 0.15 versus 1.0 ± 0.12 mol/L ∗ min, *P* = 0.003) and insulin sensitivity was decreased compared to CON (OGIS: 467.2 ± 64.1 versus 510.6 ± 53.1 mL/min ∗ m^2^, *P* = 0.01). pGDM had lower adiponectin (8.1 ± 2.6 versus 12.6 ± 5.3, *P* < 0.008) but increased waist circumference and CRP compared to CON. *Conclusions*. Despite diagnosis of normal glucose tolerance, pGDM are characterized by hyperglycemia and insulin resistance compared to healthy controls, accompanied by decreased adiponectin and increased CRP concentrations, thus linking metabolic disturbances to an increased cardiovascular risk in pGDM.

## 1. Background

Gestational diabetes mellitus (GDM) identifies women with an increased risk of developing type 2 diabetes and cardiovascular disease. Therefore, women with prior GDM (pGDM) are recommended to regularly undergo assessment of glucose tolerance [1] in order to detect overt diabetes in time to initiate treatment to prevent complications.

The current view of GDM pathophysiology is that women who develop GDM are characterized by “*beta-cell dysfunction based on chronic insulin resistance*” [2]. Consequently, disturbances in glucose metabolism are thought to be of chronic nature rather than of acute onset during pregnancy [3].

We have previously shown that pGDM with metabolic deterioration within five years after delivery are characterized by hyperglycemia, insulin resistance, and hyperinsulinemia; furthermore first-phase insulin secretion was impaired in these women compared to pGDM who did not experience deterioration of glucose tolerance [4]. In addition, while those pGDM who progress to diabetes have a marked increase in glycemia and insulin resistance before diabetes onset, we observed that beta-cell function declines continuously over years [5]. Others have shown that declining beta-cell compensation for increased insulin resistance (estimated by the disposition index) characterizes pGDM who convert to overt diabetes within 12 years after delivery [6] and that this decline was associated with an increase in body weight and C-reactive protein (CRP) as well as a decrease in adiponectin [7].

Approximately 50% of pGDM develop type 2 diabetes within the first five years after delivery [8, 9]. At the same time, there is a group of pGDM who are able to maintain normal glucose tolerance within this vulnerable period of metabolic deterioration. Although many studies describe metabolic changes in those who convert to diabetes, less is known about pGDM who are able to maintain normal glucose tolerance (NGT) over the years following delivery. It could be speculated that metabolic disturbances are cured in those pGDM and that their risk of developing diabetes equals the one of women without a history of GDM. Consequently, time- and cost-consuming follow-ups would not be necessary in these women. This could help to reduce unnecessary costs.

Since data that would answer this question are scare, the aim of this study was to compare validated parameters of insulin sensitivity and secretion between pGDM with NGT five years after gestational diabetes and healthy controls (five years after normal pregnancy). In addition, metabolic changes in pGDM within the five-year observational period and the impact of weight loss within this time were investigated.

## 2. Study Population and Methods

### 2.1. Study Population

The current study was part of the Viennese Post-Gestational Diabetes Project (VPGDP), a prospective longitudinal study in women with a history of GDM. pGDM were recruited during a pregnancy complicated by GDM in the Diabetes Outpatient Clinic of the Division of Endocrinology and Metabolsim of the Medical University of Vienna, where they had been seen during pregnancy. Healthy controls (CON) were recruited from the department of obstetrics and gynecology. The Human Ethics Committee of the Medical University of Vienna approved the protocol and all women gave written informed consent.

Exclusion criteria were age ≤ 18 years, known preexisting glucose intolerance (impaired glucose tolerance, type 1 or type 2 diabetes), diagnosis of GDM before the 8th gestational week, positive diabetes-associated antibodies (assessed during and after pregnancy at our division), ethnicity other than Caucasian, morbid obesity (pregestational body mass index > 40 kg/m^2^), or evidence of chronic diseases, including kidney or liver diseases and chronic inflammatory diseases. In CON, exclusion criteria additionally included the existence of any risk factors for diabetes (i.e., positive family history or chronic medication known to influence carbohydrate metabolism).

For the current study, 45 pGDM with normal glucose tolerance at five-year follow-up and 18 healthy controls were matched for age and body mass index (Table 1). Data from the visit 5 years postpartum were compared between the groups and in pGDM also to the baseline examination (six months after delivery). Furthermore, an additional subanalysis was performed, in which those pGDM with weight loss of ≥7% (wihtin the 5-year observational period) were compared to the healthy control group. This range was chosen in regard to the American Diabetes Association (ADA) recommendation of 7% weight loss in patients with prediabetes [10].

### 2.2. Methods

All women received dietary counseling and were recommended to regain normal body weight by intake of a healthy diet and regular physical activity. Glucose tolerance tests were scheduled between day 3 and day 10 of their menstrual cycle, performed in the morning after an overnight fast of at least 8 hours. Women were asked to refrain from physical activity 3 days prior to the follow-up visits. All women underwent an oral glucose tolerance test (OGTT) and the majority underwent also an intravenous glucose tolerance test (76% of pGDM and 61% of CON). Reasons for not undergoing an intravenous glucose tolerance test (IVGTT) were mainly problems in time scheduling (additional appointment according to menstrual cycle, more than 2 weeks after OGTT).

#### 2.2.1. Oral Glucose Tolerance Test (OGTT)

After a venous catheter was placed into an antecubital vein, blood samples for the measurement of glucose, insulin, and C-peptide were taken at fasting as well as 10, 20, 30, 60, 90, 120, 150, and 180 minutes after ingestion of 75 g glucose in a solution.

#### 2.2.2. Intravenous Glucose Tolerance Test (IVGTT)

For the IVGTT, one venous catheter for blood sampling was placed in one antecubital vein and another one for intravenous administration of glucose and insulin in an antecubital vein of the other arm. Blood samples (for measurement of glucose, insulin, and C-peptide) were drawn at the fasting state (−10 and 0 minutes) and 3, 4, 5, 6, 8, 10, 14, 19, 22, 27, 30, 35, 40, 50, 70, 100, 140, and 180 minutes after injection of glucose (300 mg/kg body weight). At 20 minutes, normal insulin (Humulin R, Eli Lilly, Indianapolis, IN, USA) was given with a concentration of 0.03 IU/kg body weight and for a duration of 5 minutes [11].

#### 2.2.3. Assessment of Metabolic Parameters, Lipids, and Cardiovascular Biomarkers

Glucose was immediately analyzed by the hexokinase method in our central lab. Serum samples for the assessment of insulin and C-peptide were immediately cooled down, centrifuged, stored at −20 degrees Celsius, and later analyzed in the lab of the Division of Endocrinology and Metabolism by commercially available radioimmunoassay kits: insulin (Serono Diagnostics, Freiburg, Germany) and C-peptide (CIS Bio International, Cedex, France) with interassay coefficients of variation of <5%.

At fasting, additional blood samples were taken and the following parameters were assessed: adiponectin was measured in duplicate by an ELISA system developed for the assessment of human plasma adiponectin concentrations (Department of Internal Medicine and Molecular Science, Osaka University, Suita, Osaka, Japan) [12]. Leptin (Human Leptin RIA kit; Linco Research, St. Charles, MO, USA) and glucagon (ICN Biomedicals, Costa Mesa, CA, USA) were measured in duplicate by commercially available radioimmunoassay kits with a CV < 6% for leptin and <8% for glucagon.

HbA1C (by high-performance liquid chromatography, given in % [13]), TSH, total cholesterol, LDL-cholesterol, HDL-cholesterol, triglycerides, and C-reactive protein [14] were measured by established methods in the Central Lab of the Medical University of Vienna.

#### 2.2.4. Data Analyses

Normal glucose tolerance (NGT) was defined according to the criteria of the ADA [10]: fasting plasma glucose (FPG) < 100 mg/dL and 2-hour post-OGTT glucose < 140 mg/dL.

The oral glucose insulin sensitivity (OGIS) index describes glucose clearance per unit change of insulin concentration [15]. Parameters of insulin secretion were described by the areas under the curve (AUC) of insulin and C-peptide during OGTT and IVGTT, calculated with the trapezoidal rule. Hepatic insulin extraction (HIE, given in %) was quantified with a mathematical model of insulin/C-peptide interactions [16].

From IVGTT, insulin sensitivity index (*S*_*I*_, in 10^−4^ min^−1^/(*μ*U/mL)) describing insulin effect on glucose disappearance [17] was computed. First-phase insulin secretion was assessed by ΔAIR_G_ calculated by averaging insulin concentrations above basal from 3 to 10 minutes and given in *μ*U/mL. The disposition index derived from IVGTT (10^−2^ min^−1^ [18]) was calculated as *S*_*I*_ × ΔAIR_G_ and describes the combined effect of insulin secretion and sensitivity on glucose disposal [19]; it is frequently used to describe the ability of the beta cells to adapt for increased insulin resistance.

#### 2.2.5. Statistical Analyses

Between group differences were calculated by ANOVA; changes between baseline and follow-up visit in pGDM were calculated by a paired *t*-test. Associations between continuous variables are described by Pearson's correlation coefficient. Data are given in means ± standard deviation. Levels of significance were set at *P* < 0.05. SAS software (Enterprise Guide 4.3, SAS Institute Inc., Cary, NC, USA) was used for all computations.

## 3. Results

### 3.1. Metabolic Differences between pGDM and CON Five Years Postpartum

**Figure 1:**
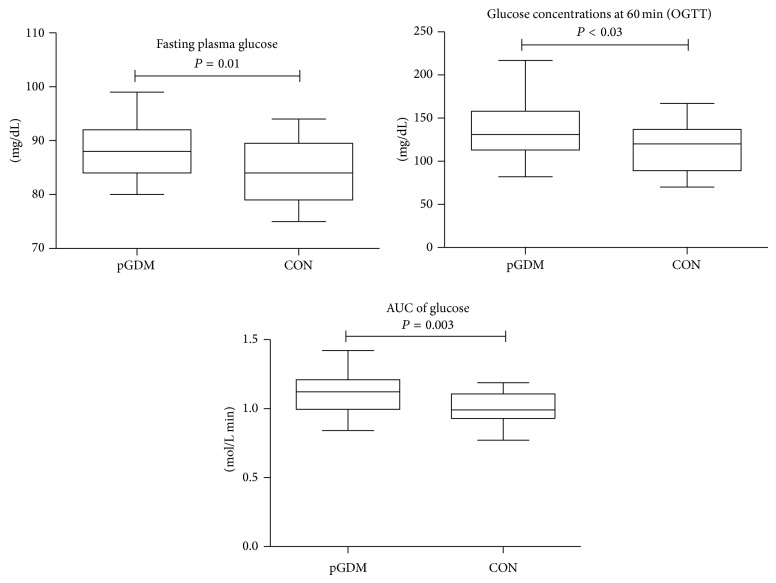
Higher glucose concentrations at fasting and stimulated conditions in pGDM compared to CON.

**Figure 2:**
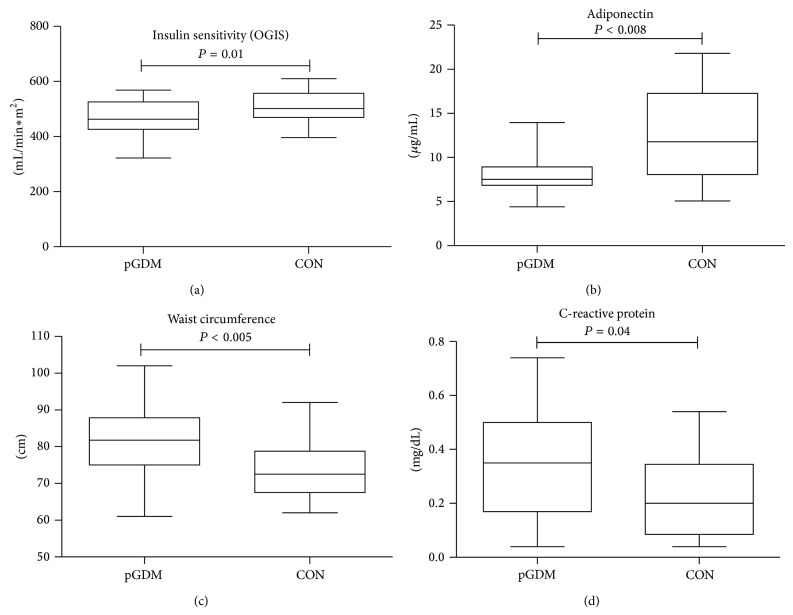
Insulin sensitivity (OGIS) and adiponectin were decreased in pGDM, while waist circumference and CRP were increased compared to CON.

Five years after the index pregnancy, pGDM—despite normal glucose tolerance—had significantly higher levels of plasma glucose at fasting as well as 60 minutes of the OGTT compared to CON; in line, the AUC of glucose was significantly increased in pGDM compared to CON (Figure 1). Insulin sensitivity, derived by OGIS, was decreased in pGDM compared to CON (Figure 2(a)). Furthermore, adiponectin was lower in pGDM (Figure 2(b)), while blood pressure, leptin, glucagon, TSH, and lipid profile did not differ between the groups. pGDM had significantly higher waist circumference (Figure 2(c)) as well as CRP concentrations, (Figure 2(d)). Normal body weight, defined as BMI < 25 kg/m^2^, was found in 30 out of 45 pGDM (=66.7%) and in 14 out of 18 CON (=77.8%). Insulin sensitivity (OGIS) was negatively correlated with BMI and body weight in the whole study group (BMI: *R* = −0.3, *P* = 0.01; body weight: *R* = −0.3, *P* = 0.02).

### 3.2. Metabolic Changes in pGDM within the Five-Year Follow-Up Period

When follow-up data in pGDM were compared to their baseline examination (= six months postpartum), waist circumference (85.5 ± 9.0 versus 81.8 ± 9.8 cm, *P* = 0.002) and diastolic blood pressure (76.7 ± 9.1 versus 72.5 ± 9.4 mmHg, *P* < 0.04) were lower at five-year follow-up compared to baseline (6 months after pregnancy). In addition, pGDM had significantly lower total cholesterol (from 211.9 ± 48.6 to 198.4 ± 40.2 mg/dL, *P* < 0.006) and LDL-cholesterol (from 134.2 ± 45.4 to 122.6 ± 37.0 mg/dL, *P* = 0.002) compared to baseline. No change was observed in body weight, HbA1C, glucagon, leptin, or TSH.

Furthermore, the disposition index (1.7 ± 1.2 versus 2.6 ± 2.3 10^−2 ^min^−1^, *P* < 0.004) and insulin sensitivity derived from IVGTT (from 4.3 ± 2.5 to 6.3 ± 3.2 10^−4 ^min^−1^/(*μ*U/mL), *P* = 0.001) were increased compared to baseline.

### 3.3. The Impact of Weight Loss on Metabolic Status at 5 Years Postpartum

Twelve pGDM had significant weight loss (≥7%) within the 5-year observational period (pGDM_wl) and were compared to CON, in order to assess whether weight loss was associated with an improved metabolic profile. While there was no difference in age, BMI, and waist circumference between the groups, pGDM_wl had significantly increased concentrations of glucose (AUC of glucose, 1.13 ± 0.16 versus 1.0 ± 0.12 mol/L ∗ min, *P* < 0.02) and insulin during the OGTT (TIS: 27.1 ± 7.1 versus 21.8 ± 5.7 nmol/L, *P* = 0.03, AUC of C-peptide: 419.5 ± 106.9 versus 347.0 ± 81.3 nmol/L ∗ min, *P* = 0.04), whereas insulin sensitivity was lower compared to CON (OGIS: 466.9 ± 46.4 versus 510.6 ± 53.1 mL/min ∗ m^2^, *P* < 0.03). Furthermore, CRP was higher in pGDM_wl compared to CON (0.4 ± 0.3 versus 0.2 ± 0.2 mg/dL, *P* = 0.04).

When pGDM_wl were compared to their baseline state, the significant changes in body weight (−9.9 ± 4.8 kg, BMI: from 26.8 ± 3.5 to 23.2 ± 2.5 kg/m^2^, both *P* < 0.0001) and waist- (from 89.0 ± 7.9 to 79.8 ± 9.4 cm, *P* = 0.0002) and hip-circumference (from 107.5 ± 5.8 to 97.8 ± 4.6 cm, *P* < 0.0001) were accompanied by an improved disposition index (from 1.6 ± 1.0 to 2.9 ± 1.6 10^−2^ min^−1^, *P* = 0.01; in line with the whole pGDM-group), a decline in CRP concentrations (from 0.6 ± 0.2 to 0.4 ± 0.3 mg/dL, *P* = 0.03), diastolic blood pressure (from 80.8 ± 9.3 to 72.2 ± 7.9 mmHg, *P* = 0.02), and leptin (from 17.7 ± 5.5 to 14.5 ± 7.0 ng/mL, *P* < 0.03).

## 4. Discussion

The current study aimed to assess whether disturbances in glucose metabolism can be observed in women with prior gestational diabetes (pGDM) who were able to maintain normal glucose tolerance (NGT) until five years after a GDM-pregnancy. According to our data, pGDM—despite normal glucose tolerance—were still characterized by decreased insulin sensitivity and increased glucose concentrations during the OGTT compared to healthy controls. Furthermore, CRP levels and waist circumference were higher in pGDM compared to CON, despite comparable BMI, while adiponectin was decreased in pGDM. In addition, pGDM with weight loss ≥ 7% within the five-year follow-up period exhibited pronounced metabolic disturbances compared to CON.

Insulin resistance is a frequent finding in women with prior gestational diabetes and associated with ectopic lipid accumulation in skeletal muscle and liver [20, 21]. It is assumed that gestational diabetes develops on the background of chronic insulin resistance, aggravated by the physiological insulin resistance of late pregnancy [2, 3]. According to our observations, we also assume that insulin resistance in pGDM is of chronic nature and the diagnosis of GDM during pregnancy detects a metabolic phenotype with increased insulin resistance in a young female cohort.

We found a weak, but significant, inverse association between BMI and insulin sensitivity; however, also in the subgroup of pGDM with significant weight loss (pGDM_wl) insulin sensitivity was significantly lower compared to the healthy control group. Special attention has to be given to this subgroup of pGDM who had significant weight loss of ≥7% within the follow-up period. This group had a BMI of approximately 27 kg/m^2^ six months after delivery and five years postpartum mean BMI was 23.2 ± 2.5 kg/m^2^. Weight loss in this group was accompanied by a reduction in waist- and hip-circumferences as well as leptin; furthermore, the disposition index (in line with the whole study group) improved; however, despite marked changes in body weight, insulin sensitivity and glucose concentrations during the OGTT and IVGTT remained unchanged compared to the baseline examination (6 months postpartum). Hence, it might be speculated that obesity is not the main reason for insulin resistance in pGDM. This assumption is supported by one of our prior observations, which showed that insulin resistance is pronounced in lean subjects with GDM and persists after delivery [22]. In addition, it is in line with prior investigations in women with a history of GDM showing that the decline in insulin sensitivity and beta-cell compensation could not be explained by changes in adiposity [23]. It has also been shown that impaired beta-cell glucose sensitivity independent of obesity and hyperglycemia displays a risk factor in pGDM [24].

It has to be noted that the majority of women in our study groups (66.7% in pGDM and 77.8% in CON) had a BMI lower than 25 kg/m^2^ and thus fulfill the criteria of normal body weight. Furthermore, mean waist circumference in pGDM was 81.8 cm, which would fulfill the WHO criteria for metabolic syndrome, but not those of the US National Cholesterol Education Program Adult Treatment Panel III [25]. This again leads us to conclude that obesity might not be the main trigger of glucose intolerance in this young female cohort or not in all pGDM.

The observation that glucose concentrations during the OGTT were higher in pGDM compared to CON clearly indicates a risk of hyperglycemia in these women. This risk would not have been detected by simply concentrating on the definition of NGT, because glucose values were within the normal range; however, in comparison to age- and BMI-matched controls, glucose concentrations during the OGTT were significantly higher in pGDM. And even in pGDM_wl, the significant weight loss did not counteract increased glucose concentrations compared to CON.

The finding that adiponectin was decreased and CRP increased in pGDM compared to CON is of great interest. A recent study by Xiang and coworkers [6, 7] described that declining beta-cell compensation in pGDM (described by the disposition index) is—besides weight gain—associated with declining levels of adiponectin and rising CRP levels. To our surprise, these metabolic features were also observed in our group of pGDM who were able to maintain normal glucose tolerance within this vulnerable period of five years postpartum. Hence, the question of whether these metabolic alterations can be used as markers of metabolic deterioration or simply reflect this special metabolic profile in pGDM appears. Specifically a drop in adiponectin could reflect metabolic deterioration in pGDM and indicate the need for closer follow-up of these women; however, this assumption has to be reexamined in future prospective studies.

Chronic inflammation and hypoadiponectinemia are frequently found in patients with diabetes; hence it appears that these disturbances could be the cause rather than the consequences of insulin resistance and hyperglycemia. In addition, this combination of hypoadiponectinemia and increased CRP might contribute to the development of atherosclerosis [26, 27]. Hence, we could assume that pGDM despite normal glucose tolerance have an increased cardiovascular risk and should be considered as high-risk population for cardiovascular disease.

Several metabolic parameters, that is, LDL-cholesterol, diastolic blood pressure, and waist circumference as well as the disposition index and insulin sensitivity derived from IVGTT, were improved in pGDM at five years postpartum compared to baseline. But despite these ameliorations and the fact that the majority of our pGDM group were able to regain/maintain normal body weight and normal glucose tolerance, disturbances in glucose regulation were observed. Hence, it appears that the metabolic profile and thus the risk of developing type 2 diabetes seem to be chronic and that GDM only identifies women at risk. It appears that the pivotal mechanisms that finally lead to the development of overt hyperglycemia in pGDM have not been elucidated in detail. This may include a genetic disposition in this group of GDM without obesity but still increased risk for type 2 diabetes. As shown, GDM risk is associated with genes involved in the regulation of insulin secretion [28]. At least, more studies are needed to better understand the development of overt hyperglycemia and develop treatment strategies, which can improve prevention.

While the strength of the current study lies in the investigation of a well-characterized cohort and performance of validated tests under dynamic—not only fasting—conditions, it also has some limitations: the study group is quite small and follow-up is limited to five years; an extended follow-up period could allow strengthening our conclusions.

Consequently we can summarize that metabolic disturbances which predispose pGDM to the development of overt diabetes appear to be chronic and can be hidden but, still, remain life-long and therefore regular follow-ups should be recommended to all women with a history of GDM in order to detect diabetes in time and prevent complications, especially the onset of cardiovascualr disease.

## Figures and Tables

**Table 1:** Baseline characteristics in women with prior gestational diabetes (pGDM) and healthy controls (CON).

	pGDM	CON	*P* value
Age (years)	37.0 ± 4.1	35.2 ± 5.3	ns
Body mass index (kg/m^2^)	24.3 ± 3.1	23.3 ± 3.3	ns
Waist circumference (cm)	81.8 ± 9.8	73.6 ± 8.3	<0.005
HbA1C (%)	5.3 ± 0.3	5.3 ± 0.2	ns
Fasting plasma glucose (mg/dL)	88.2 ± 5.6	84.0 ± 6.2	0.01

## References

[1] American Diabetes A (2013). Standards of medical care in diabetes. *Diabetes Care*.

[2] Metzger B. E., Buchanan T. A., Coustan D. R. (2007). Summary and recommendations of the Fifth International Workshop-Conference on Gestational Diabetes Mellitus. *Diabetes Care*.

[3] Buchanan T. A., Xiang A. H. (2005). Gestational diabetes mellitus. *The Journal of Clinical Investigation*.

[4] Winhofer Y., Tura A., Prikoszovich T. (2013). The impact of recurrent gestational diabetes on maternal metabolic and cardiovascular risk factors. *European Journal of Clinical Investigation*.

[5] Tura A., Grassi A., Winhofer Y. (2012). Progression to type 2 diabetes in women with former gestational diabetes: time trajectories of metabolic parameters. *PLoS ONE*.

[6] Xiang A. H., Kjos S. L., Takayanagi M., Trigo E., Buchanan T. A. (2010). Detailed physiological characterization of the development of type 2 diabetes in hispanic women with prior gestational diabetes mellitus. *Diabetes*.

[7] Xiang A. H., Kawakubo M., Trigo E., Kjos S. L., Buchanan T. A. (2010). Declining *β*-cell compensation for insulin resistance in hispanic women with recent gestational diabetes mellitus: association with changes in weight, adiponectin, and C-reactive protein. *Diabetes Care*.

[8] Metzger B. E., Cho N. H., Roston S. M., Radvany R. (1993). Prepregnancy weight and antepartum insulin secretion predict glucose tolerance five years after gestational diabetes mellitus. *Diabetes Care*.

[9] Kim C., Newton K. M., Knopp R. H. (2002). Gestational diabetes and the incidence of type 2 diabetes: a systematic review. *Diabetes Care*.

[10] American Diabetes Association (2014). Standards of medical care in diabetes—2014. *Diabetes Care*.

[11] Pacini G., Tonolo G., Sambataro M. (1998). Insulin sensitivity and glucose effectiveness: minimal model analysis of regular and insulin-modified FSIGT. *The American Journal of Physiology—Endocrinology and Metabolism*.

[12] Stefan N., Vozarova B., Funahashi T. (2002). Plasma adiponectin concentration is associated with skeletal muscle insulin receptor tyrosine phosphorylation, and low plasma concentration precedes a decrease in whole-body insulin sensitivity in humans. *Diabetes*.

[13] Goldstein D. E., Little R. R., Wiedmeyer H. M., England J. D., McKenzie E. M. (1986). Glycated hemoglobin: methodologies and clinical applications.. *Clinical chemistry*.

[14] Dowton S. B., Colten H. R. (1988). Acute phase reactants in inflammation and infection. *Seminars in Hematology*.

[15] Mari A., Pacini G., Murphy E., Ludvik B., Nolan J. J. (2001). A model-based method for assessing insulin sensitivity from the oral glucose tolerance test. *Diabetes Care*.

[16] Tura A., Ludvik B., Nolan J. J., Pacini G., Thomaseth K. (2001). Insulin and C-peptide secretion and kinetics in humans: direct and model-based measurements during OGTT. *The American Journal of Physiology—Endocrinology and Metabolism*.

[17] Pacini G., Bergman R. N. (1986). MINMOD: a computer program to calculate insulin sensitivity and pancreatic responsivity from the frequently sampled intravenous glucose tolerance test. *Computer Methods and Programs in Biomedicine*.

[18] Pacini G. (2006). The hyperbolic equilibrium between insulin sensitivity and secretion. *Nutrition, Metabolism and Cardiovascular Diseases*.

[19] Ahrén B., Pacini G. (2004). Importance of quantifying insulin secretion in relation to insulin sensitivity to accurately assess beta cell function in clinical studies. *European Journal of Endocrinology*.

[20] Prikoszovich T., Winzer C., Schmid A. I. (2011). Body and liver fat mass rather than muscle mitochondrial function determine glucose metabolism in women with a history of gestational diabetes mellitus. *Diabetes Care*.

[21] Kautzky-Willer A., Krssak M., Winzer C. (2003). Increased intramyocellular lipid concentration identifies impaired glucose metabolism in women with previous gestational diabetes. *Diabetes*.

[22] Kautzky-Willer A., Prager R., Waldhäusl W. (1997). Pronounced insulin resistance and inadequate *β*-cell secretion characterize lean gestational diabetes during and after pregnancy. *Diabetes Care*.

[23] Xiang A. H., Takayanagi M., Black M. H. (2013). Longitudinal changes in insulin sensitivity and beta cell function between women with and without a history of gestational diabetes mellitus. *Diabetologia*.

[24] Tura A., Mari A., Winzer C., Kautzky-Willer A., Pacini G. (2006). Impaired *β*-cell function in lean normotolerant former gestational diabetic women. *European Journal of Clinical Investigation*.

[25] Grundy S. M., Brewer H. B., Cleeman J. I., Smith S. C., Lenfant C. (2004). Definition of metabolic syndrome: Rep ort of the National Heart, Lung, and Blood Institute/American Heart Association conference on scientific issues related to definition. *Circulation*.

[26] Ouchi N., Kihara S., Funahashi T., Matsuzawa Y., Walsh K. (2003). Obesity, adiponectin and vascular inflammatory disease. *Current Opinion in Lipidology*.

[27] Matsubara M., Namioka K., Katayose S. (2003). Decreased plasma adiponectin concentrations in women with low-grade C-reactive protein elevation. *European Journal of Endocrinology*.

[28] Zhang C., Bao W., Rong Y. (2013). Genetic variants and the risk of gestational diabetes mellitus: a systematic review. *Human Reproduction Update*.

